# Instruments that measure evidence-based practice knowledge, skills, and attitudes among health professions students: A systematic review protocol

**DOI:** 10.1371/journal.pone.0347078

**Published:** 2026-07-13

**Authors:** Danill Stolear, Lynne M. Z. Lafave, Breda Eubank, Mark R. Lafave

**Affiliations:** 1 Department of Psychology, Faculty of Arts, Mount Royal University, Calgary, Alberta, Canada; 2 Department of Health and Physical Education, Faculty of Health, Community, and Education, Mount Royal University, Calgary, Alberta, Canada; Banasthali Vidyapith, INDIA

## Abstract

**Background:**

Evidence-based practice (EBP) is recognized as a foundational element of health professional education worldwide. The Sicily Statement is a five-step model for both clinical practice and teaching EPB: Ask, Acquire, Appraise, Apply, Audit (or evaluate) [Dawes M, et al. BMC Med Educ. 5(1):1 (2005)]. Evaluating EBP competence in health professions’ students is critical to develop strategies that build that competence and ultimately lead to optimal patient care.

**Rationale:**

There are many EBP measurement instruments that have been identified in previous reviews, but none that were intended for the health professions student population [Tilson JK, et al. BMC Med Educ. 11:78 (2011)].

**Objective:**

This systematic review aims to summarize and evaluate the measurement properties of existing instruments that evaluate health professions students’ EBP competence including EBP knowledge, skills, and attitudes.

**Methods:**

The systematic review was registered with PROSPERO under the identifier CRD42024564908. This systematic review will follow an adapted version of the eight-step process for systematic reviews for patient-reported outcome measures (PROMs)(3). The adaptations will align with the research question and EBP measurement instruments that are the primary target of this review. The search strategy will employ key words and phrases from the objective targeting following electronic databases: MEDLINE, CINAHL, SPORTDiscuss, and PsycINFO, all via EBSCO. Nine psychometric measurement properties will act as the foundation for data extraction and evaluation of the measurement instruments. Data will be extracted and risk of bias will be completed on an individual study basis and on an EBP measurement instrument basis. A unique GRADE reporting system will be employed that accounts for a single conclusion based on a composite of the nine measurement properties and synthesized results from all studies.

## Introduction

Evidence-based practice (EBP) is recognized as a foundational element of health professional education worldwide [1,2]. Conceptually, EBP has been around since the 1960’s, but it was more formally articulated as evidence-based medicine (EBM) by Guyatt et al. in 1992. Since then, the EBP framework has continued to evolve considerably. One of the most notable developments has been the shift in terminology from EBM to EBP.

Dawes et al. developed the Sicily Statement that defined EBP as *“[practice that] requires decisions about health care that are based on the best available, current, valid and relevant evidence* [3]*. These decisions should be made by those receiving care, informed by the tacit and explicit knowledge of those providing care, within the context of available resources*” [3, pg. 4]. Dawes et al. also developed a five-step model for both clinical practice and teaching EPB: Ask, Acquire, Appraise, Apply, Audit (or evaluate) [3]. Tilson et al. went on to develop a CREATE framework that would merge this five-step model with how to measure EBP with various assessment strategies, including, but not limited to assessment instruments like those central to this study [4].

Bloom et al. was one of the first to use the knowledge, skills, and attitudes framework to help parse the components of competence [5]. This same knowledge, skills, and attitudes framework has been subsequently applied in healthcare education [6,7]. Mills et al. defined competence as “the observable ability of a person, integrating knowledge, skills, and attitudes in their performance of tasks” [6, pg. 12].

Shaneyfelt et al. were one of the first groups to complete a systematic review that searched for EBP instruments, with a focus on measuring the effectiveness of medical educational strategies [8]. They concluded that more development and testing was needed to measure EBP attitudes, behaviors, and skills. Shaneyfelt et al., however, did not include knowledge as a measure of EBP. Neither did Landsverk et al. who completed a similar systematic review of EBP in health professionals. Landsverk et al. also adopted and adapted the Consensus-Based Standards for the Selection of Health Measurement Instruments (COSMIN) methodology for systematic reviews for patient reported outcome measures in quality assessment, rating, and quality of evidence on the development and content validity of each study/tool [9,10]. Haavisto et al. completed a scoping review to assess instruments that measured healthcare professionals’ EBP knowledge and skills [11]. However, they did not include attitudes or self-efficacy.

Although much research has been done around EBP in health professions, there is an opportunity to explore and evaluate which instruments measure health professions students’ EBP competence including EBP knowledge, skills, and attitudes in health undergraduate or graduate students. Several instruments have been developed to evaluate and measure EBP knowledge, skills, and attitudes in this population. Not all instruments have been quality-rated, nor have its measurement properties been assessed. Additionally, no consensus has been reached on which instrument is most suited for measuring EBP constructs or competence in health professions, let alone for students.

### Objectives

This systematic review aims to summarize and evaluate the measurement properties of existing instruments that evaluate health professions students’ EBP competence including EBP knowledge, skills, and attitudes. This study will build on Landsverk et al. and describe in detail steps on how to adapt the COSMIN methodology for EBP instrument quality assessment using the updated version of the COSMIN guidelines [9,10]. Landsverk et al were the first to employ the COSMIN guidelines, which was a ten-step process at the time [9,10]. The updated COSMIN guidelines for systematic reviews have moved to an eight-step process.

Specifically, this systematic review will answer the following guiding research question: In health professional students (P), what instruments (I) demonstrate valid and reliable measurement properties for assessing EBP competence (O) including the subscales of knowledge, skills (including intention to practice, behaviours, competency, and patient benefits), and attitudes (including confidence, self-efficacy, beliefs and/or motivation)?

## Methods

This systematic review will be conducted and reported according to the Preferred Reporting Items for Systematic Reviews and Meta-Analyses (PRISMA) 2020 checklist [12]. Additionally, the COSMIN guidelines for systematic reviews (COSMIN MSR) of Patient Reported Outcome Measures (PROMs) will be adapted for synthesizing evidence, quality assessment, and rating (i.e., content validity) of existing EBP instruments that can be used in clinical practice and to teach health professions students, similar to what Landsverk et al. completed for practicing health professions [9,10]. The COSMIN MSR is being employed because it guides a process that accounts for the nine measurement properties for measurement instruments, which is a unique study relative to the original intended purpose of systematic reviews with observational studies or randomized control trials.

Conducting systematic reviews of EBP measurement instruments employs an eight-step process when drawing a conclusion on each instrument [10]. Therefore, we will outline how to adapt the COSMIN MSR for PROMs for the systematic review of EBP tools for health professions’ students. We will follow the procedures outlined by COSMIN MSR closely, but we will outline the exact method that will be employed below and identify any variation from the previously published guidelines.

The first four steps follow a standard process for systematic reviews. Steps 5–7 are where many of the adaptations will occur. Again, there is complexity of this systematic review due to the fact there are nine measurement properties that COSMIN MSR has identified that need to be accounted for in all measurement instruments to make a recommendation or conclusion about the instruments’ psychometric soundness. However, the COSMIN MSR also employs a ten-item risk of bias (ROB) checklist that overlaps precisely with the 9 measurement properties but adds “PROM development” as a first step in assessing the quality of the study. These are two separate assessments, but the psychometric rating will help to inform the ROB ratings. Step 6 is the most complicated component of this systematic review and where our methods differ the most from COSMIN MSR. It involves psychometric measurement property rating and ROB for individual studies. Then, where there is more than one study for a specific EBP measurement instrument, we will combine both the psychometric measurement property ratings and ROB assessment, which considers many variables and thus complicates the ability to make a simple and singular recommendation. As a result, we will introduce a unique method to account for the summary of the ROB assessment roughly following a GRADE approach in Step 6 and 7 below. Step 8 is merely writing and submitting the review.

### Criteria for considering studies for this review

1) **Formulate your research question**

The systematic review was registered with PROSPERO under the identifier CRD42024564908 (Appendix A in S1 Appendix).

2) **Formulate eligibility criteria**

A complete list of inclusion and exclusion criteria are listed in Appendix B in S2 Appendix, but we will include a general overview of the inclusion and exclusion criteria below.

#### Types of studies.

Studies must be peer-reviewed, measure healthcare professionals’ self-reported attitudes (including confidence, self-efficacy, beliefs and/or motivation), and/or skills (including intention to practice, behaviours, competency, and patient benefits). Studies that only measure knowledge will not be included as they are deemed to only measure one component of EBP competence. We will include primary studies that developed an EBP tool or have employed an EBP tool in a study that adds to the nine measurement properties recognized as important from a developmental perspective for the tool.

#### Types of participants.

Students enrolled in post-secondary studies within healthcare professional programs. Disciplines outside of healthcare-related fields that utilize EBP will be excluded, such as, but not limited to, primary or secondary school teachers.

#### Types of outcomes.

Inclusion criteria for this study were the measurement of EBP attitudes and/or skills. Measurements of EBP knowledge were included only if there was also a measurement of attitudes and/or practice. For example, some studies only measured a combination of knowledge and attitudes, knowledge and skills, or skills and attitudes, all of which would be eligible for inclusion. Many studies only focused on the measurement of knowledge, so if that were the case, these studies will not be included. To be considered for inclusion, studies needed to report on the 10 “boxes” outlined by risk of bias items 6.2 below.

#### Types of interventions.

This review does not include any criteria for interventions.

#### Primary outcome.

The primary outcome of this review will be to evaluate EBP measurement instruments that measure EBP competence, including its subscales of knowledge, skills, and attitudes.

#### Timeline.

All historical studies that meet all the inclusion and exclusion criteria will be considered up to and including May 15, 2026.

### Electronic searches

3) **Develop a literature search**

The search strategy will aim to locate published studies on the measurement of EBP competence and its subscales. A three-step search strategy will be utilized in this review. First, an initial limited search of MEDLINE (EBSCO) and CINAHL (EBSCO) will be undertaken to identify articles on the topic. This search will identify several reviews that are like the current review objective but did not focus on students [9,1,11,13,14]. Inclusion and exclusion criteria from these reviews relevant to students will inform the criteria for this review. Additionally, we have identified key test articles that we have confirmed meet our search criteria and study EBP instruments intended for health professions’ students [15,16].

This review will search MEDLINE, CINAHL, SPORTDiscuss, and PsycINFO, all via EBSCO. SPORTDiscuss will locate any articles not caught by MEDLINE and CINAHL. PsycINFO will locate any articles that investigate instruments in social professions where EBP may also be utilised. ERIC will not be included due to the lack of relevance to our research question.

#### Searching other sources.

If there are non-English studies, language experts will be brought into the team as collaborators to determine if they meet inclusion and exclusion criteria. Dissertations, conference abstracts, editorials, case reports, and non-primary research (reviews and meta-analyses) will be excluded. Those studies that measure all the subscales of the EBP construct will be included (i.e., knowledge, skills, and attitudes). We will also include individual or combinations of those subscales with one exception. Instruments that only measure the EBP knowledge subscale will be excluded. We will hand search studies based on cited literature of included studies.

A copy of the search strategy is in Appendix C in S3 Appendix.

### Data collection and analysis

4) **Conduct a literature search**

Articles that meet our search criteria will be uploaded into Covidence software (*Covidence*, n.d.). All the reviewers will be blinded to the inclusion and exclusion decisions of the others and complete screening on their own time to reduce bias risk. Two reviewers (all authors) will screen the titles and abstracts of the articles for inclusion and exclusion criteria prior to moving onto the full-text screening phase. All conflicts will be resolved by a third reviewer (one of the authors) who will not be involved in title and abstract screening to reduce bias. For articles that are written in non-English languages, we will bring a collaborator to assist with the screening process. The articles that make it into the full-text screening phase will be uploaded to Covidence in their full-text format and will be screened for inclusion criteria by four reviewers (all authors). Votes from two reviewers will be necessary to include or exclude articles. Conflicts will be resolved via consensus from all four reviewers. We will use the following conflict resolution criteria and code it as such in Covidence:

1. No Full text article available
2. Not primary source journal article
3. Not relevant
4. EBP concepts missing or peripheral
5. Sample is not student health profession
6. Lacks psychometric property or instrument validation discussion
7. Knowledge evaluation only

### Selection of studies

5) **Organize the studies from your articles**

Mokkink et al. recommend an initial data extraction process to determine which instruments to include in the review or not (3, pg. 2931). Our review will follow this step, generally, but we have outlined a more detailed process guided by the Appendix D in S4 Appendix template. Two members of our research team (DS and ML) will extract the relevant data from each individual study to evaluate instruments that measure EBP competence (or its subscales) in a student population. The template has one tab each and we will outline instructions of how we will use it:

### Data extraction and management

The data collected in this tab will include the EBP measurement instrument that is being studied, the authors, the EBP subscale construct(s) being studied (KSA), a study classification/purpose (i.e., to validate, translate, or originally develop), the country/language of the EBP instrument, if it is a uni-dimensional (i.e., EBP construct) or multi-dimensional (EBP subscales), the student population discipline, and the sample size.

We will pilot test this data extraction method with our authors (all four authors) using the template with four studies. Once the initial pilot testing is complete, two authors (DS and ML) will extract all data independently using their own template that is populated with the studies that have been included. If there is a discrepancy in data extraction between the authors, the two will meet to resolve the conflict. If they are unable to resolve the conflict, a third or fourth author (LL or BE) will break the tie.

6) **Evaluate the quality of the EBP competence measurement instrument**

Mokkink et al. built on their previous work from 2018 whereby the quality evaluation was broken into six sub-steps (3, pg. 2933). Again, due to differences in the purpose from Mokkink et al. (PROMs evaluation for patients) and our review (student EBP competence instrument evaluation), we will expand on where we align and where we differ in more detail below [10]. We have created a template (Appendix E in S5 Appendix) that will be used for sub-steps 6.1, 6.2, and 6.3. In brief, Steps 6.1, 6.2, and 6.3 are related to extracting data, completing a psychometric measurement property rating of the evidence against the nine criteria for good measurement properties, and completing a risk of bias assessment on an individual study level. Steps 6.4, 6.5 and 6.6 are similar, but the psychometric measurement property rating and risk of bias assessment are done based on stratifying data by EBP measurement instrument. All sub-steps in Step 6 will be done by two independent raters (DS and ML). Where there is disagreement on the rating, discussion will ensure consensus. If they are unable to resolve the conflict, a third or fourth author (LL or BE) will break the tie.

**Step 6.1 – Extract data on study populations, methods, and results**

#### Measurement property data extraction.

The data collected in this tab will include the measurement property criteria that will assist in determining the quality of a study and of an EBP instrument. These include: whether the authors clearly define how the EBP instrument was developed or they reference an original article that defines it; content validity; structural validity; internal consistency; cross-cultural validity; reliability; measurement error; criterion validity; hypothesis testing or construct validity; responsiveness. Relevant data for each paper will be added to the “reason/comment” cells for each measurement property/box.

### Assessment of risk of bias in included studies

**Step 6.2 – Assess the methodological quality of the studies – is there risk of bias?**

There is an overlap in the psychometric measurement property rating -aka from Mokkink et al. as “criteria for good measurement quality” – and the ROB assessment [10]. Therefore, we will collect data for Step 6.2 and 6.3 in the same template (Appendix E in S5 Appendix). Step 6.2 is a ROB assessment, and we will generally follow the same criteria set out by Mokkink et al., Appendix 1, the ROB Checklist 3.0 [10]. It consists of 10 boxes, each representing measurement properties of a EBP competence measurement instrument, plus the EBP instrument development item. The ROB rating scale consists of the following classifications: very good; adequate; doubtful; and inadequate. The variables that go into these classifications are outlined in detail in Mokkink et al.’s Appendix 1 and we will follow those classification criteria, with a few exceptions that we will specify below as well as in our template (Appendix E in S5 Appendix).

There is a summary tab in Appendix E in S5 Appendix that will capture the independent ratings for each individual study, broken down by boxes that are in their own separate tabs. Each rater (DS and ML) will read the paper and provide their independent assessment for each criterion outlined in detail in the specific box. If there is consensus, one author will translate the overall rating into the “methodological quality” assessment in the “Ind. Study ROB & Psych. Rating” tab. If they are unable to resolve the conflict, a third or fourth author (LL or BE) will break the tie.

**Box 1. EBP Instrument Development**

Mokkink et al. recommend 22 items to assess EBP instrument development (3, Appendix 1). However, there is a significant focus on patient interviews since their instrument is intended to measure patient-reported outcomes. In comparison, the EBP instrument development does not require so much patient consultation, so we have included five key criteria in the Tab title “Box 1.” Two raters (DS and ML) will read the article, list the corresponding Covidence identifier, and rate each question independently. Where there is no consensus, a discussion will ensue after re-reading the article and final agreement will be made. If they are unable to resolve the conflict, a third or fourth author (LL or BE) will break the tie. This process will be repeated for all studies that are listed in the summary tab. The ROB assessment scale (i.e., very good, adequate, doubtful, inadequate, or not applicable) will be associated with a numerical value (i.e., 4, 3, 2, 0, n/a, respectively) and the final mean value will be transferred to the summary tab document. Conversion from a label (i.e., very good) to a numerical value was done to simplify these metrics when they are summarized and stratified by EBP measurement instrument (i.e., Step 6.6 below). We work under the assumption that there is most likely one EBP instrument development study (i.e., the original), but if there are more, we will merely add more studies to the template.

**Box 2. EBP Instrument Content Validation Studies**

The Box 2 tab in this template is intended for studies that measure content validity. This may or may not be the original study of interest. There is space for more than one study in Box 2 since there may be more than one study that reported content validity measures. The original Box 2 in the Mokkink et al. article lists a total of 42 items to assess this measurement property (3, Appendix 1). Our template reduces this to 12 items because there is no consultation with patients, rather we only ask about relevance and comprehensiveness to professionals who teach and measure EBP competence. We have also added two other comprehensibility metrics that were not part of the COSMIN MSR: 1) Are there no floor or ceiling effects?; where V = very good or #4; I = inadequate or #0; 2) Is the reading level of the tool appropriate for the population of interest? V = very good because the reported between grade 6–12 reading level; or #4; A = adequate because they reported grade reading level, but was higher than 12 or #3; D = Doubtful because they reported grade reading level too high or too low or 2; I = inadequate or #0 because they did not report grade reading level. There are no standards for this metric in the literature, so our metrics were based on previous literature that measured reading levels in patient-reported outcome measures [17].

**Box 3. Structural Validity**

All Mokkink et al. ROB criteria will be employed [10].

**Box 4. Internal Consistency**

All Mokkink et al. ROB criteria will be employed [10].

**Box 5. Cross-cultural Validity**

All Mokkink et al. ROB criteria will be employed except one because it was not relevant to EBP measurement instruments: “Patients stable on construct between measurements” (3, Appendix 1).

**Box 6. Reliability**

This was a box that was changed significantly to target the EBP competency construct that we are studying. This box was changed from 8 items in the Mokkink et al. ROB rating to 5 items [10]. Additionally, the five items that were retained were all edited to account for the three subscales we are interested in measuring for EBP competence: knowledge, skills, and attitudes. We specified the test-retest reliability and KR 20 should be completed with a reliability metric greater than 0.70. Additionally, the recall period was changed with specific standards that are acceptable.

**Box 7. Measurement Error**

This box was changed to target the EBP competency construct that we are studying. This box was changed from 6 items in the Mokkink et al. ROB rating to 4 items [10]. The first item that was removed was related to patient stability, which is irrelevant to our study and population of interest. The second item removed is related to percent agreement; again, not related to our topic. The remaining items’ wording will stay the same.

**Box 8. Criterion Validity**

This was a box that was changed to target the EBP competency construct that we are studying. This box was changed from 3 items in the Mokkink et al. ROB rating to 2 items [10]. The one item that was removed was related to sensitivity and specificity, and therefore not applicable to our study.

**Box 9. Hypothesis Testing**

All Mokkink et al. ROB criteria will be employed [10].

**Box 10. Responsiveness**

This was a box that was changed to target the EBP competency construct that we are studying. This box was changed from 13 items in the Mokkink et al. ROB rating to 6 items [10]. Two entire subsections of Box 10 were eliminated. The “criterion approach” seemed to repeat the items in Box 8. The “construct comparator” was eliminated because there is no comparator for the EBP measurement construct and the instruments we are studying are self-reported. The remaining items from the two subsections (construct comparison between groups and construct comparison before and after intervention) were directly relevant to our review and all the items were retained.

**Step 6.3 Rate the study results against criteria for good measurement properties**

We will use the same template (Appendix E in S5 Appendix) as was used in Step 6.2. Just like Step 6.2, there is a summary tab (entitled “Ind. Study ROB & Psych. Rating”) where psychometric property measurement rating data will be entered based on the mean assessment of each individual box tab. The psychometric property measurement rating is the same as the “criteria for good measurement properties” in Mokkink et al.’s Table 1, with a few minor exceptions (3, pg. 2935). The criteria that guide the rating are at the bottom part of the tabbed spreadsheet. In other words, the ROB assessment criteria are at the top of Box 2, and the psychometric measurement rating scale is at the bottom. This same process will be followed for Boxes 2–10 inclusively. It is important to remember that Box 1 is only rated for ROB assessment. The rating criteria for each of the nine psychometric measurement properties are otherwise the same as what Mokkink et al. have recommended (3, Table 1). The rating scale that Mokkink et al. employed will be converted to a numerical value so that summarizing more than one article is easier to consolidate and understand. Therefore, the + = sufficient will be assigned a value of 2 points, the - = insufficient will be assigned a value of zero, and the? = indeterminate will be assigned a value of 0 or 1 depending on the rater’s judgement.

**Table 1:** Conversion of numerical values from overall methodological quality to standard GRADE definitions.

GRADE Definition	GRADE Explanation [18]	Guideline for Methodological Quality Recommendation
High quality	Further research is very unlikely to change our confidence in the estimate of effect	Overall methodological quality values between 3–4 and an overall mean psychometric measurement property rating between 1–2
Moderate quality	Further research is likely to have an important impact on our confidence in the estimate of effect and may change the estimate	Overall methodological quality values between 2–3 and an overall mean psychometric measurement property rating between 0–1
Low quality	Further research is very likely to have an important impact on our confidence in the estimate of effect and is likely to change the estimate	Overall methodological quality values between 1–2 and an overall mean psychometric measurement property rating between 0.5–1
Very low quality	Any estimate of effect is very uncertain	Overall methodological quality values between 0–1 and an overall mean psychometric measurement property rating below 0.5

### Measures of treatment effects, units of analysis, data synthesis, assessment of reporting biases

**Step 6.4 Summarize the results**

The previous steps in Step 6 have been related to extracting data, the ROB assessment and rating the study against good measurement property criteria for individual studies. There may be more than one study on a specific EBP measurement instrument. In these cases, we will combine data in a new template (Appendix F in S6 Appendix) and summarize the results. We will simply copy and paste data from the summary tab (entitled “Ind. Study ROB & Psych. Rating”) in Appendix E in S5 Appendix, and we will stratify the data by EBP measurement instrument so that we can summarize the results for each EBP measurement instrument. Therefore, we will sort by the EBP measurement instrument in Appendix E in S5 Appendix, then cut and paste into Appendix F in S6 Appendix. There is also a tab for single study EBP instruments in Appendix F in S6 Appendix and data from Appendix E in S5 Appendix can also be transferred. Clearly, if there is only one study, the mean values obtained from Appendix E in S5 Appendix will be the value that is used for Step 7 below.

**Step 6.5 Rate the summarized results against criteria for good measurement properties**

The same process outlined in Step 6.4 will move the “psychometric measurement property rating” over from Appendix E to Appendix F in S5 and S6 Appendix after the data has been sorted by EBP instrument. Once again, where appropriate, there may be more than one study on a specific EBP measurement instrument. In these cases, we will combine data in a new template stratified by EBP measurement instrument. Single-study EBP instruments will be captured on a different tab, and mean values will be taken from that study only. The EBP instruments that have more than one study will use the mean score across all studies. The overall mean score will be calculated on all nine psychometric measurement properties, and this score will feed into Step 7.

**Step 6.6 Grading the quality of the evidence – summary ROB assessment**

The same process outlined in Step 6.4 will move the “methodological quality assessment” over from Appendix E to Appendix F S5 and S6 Appendix and the data will be sorted by EBP instrument. Once again, where appropriate, there may be more than one study on a specific EBP measurement instrument. In these cases, we will combine data in a new template stratified by EBP measurement instrument. Single-study EBP instruments will be captured on a different tab, and mean values will be taken from that study only. The EBP instruments that have more than one study will use the mean score across all studies. Mokkink et al. recommend the GRADE approach in this step to “grade the certainty of evidence” (3, pg. 2936). However, they only explain how to GRADE the first two boxes (EBP Instrument Development and Content Validity). Our approach will be very different because we will attempt to factor in all ten boxes in our grading of the certainty of evidence for EBP instruments. We will use both the individual box mean scores, and the mean scores across all 10 boxes because we value all boxes in the final assessment. The overall mean score will be calculated on all 10 boxes, and this score will feed into Step 7. We agree with Mokkink et al.’s stance that without strong values in both Box 1 (EBP Instrument Development) and Box 2 (Content Validity), the evidence from the rest of the boxes (i.e., Box 3–10) is significantly less important and will therefore result in a lower grade in Step 7.

7) **Drawing conclusions**

We will use the original GRADE categories as a basis to draw conclusions on the summary of the evidence across 10 boxes and perhaps multiple studies that have contributed to the amassed evidence. We will link the numerical values from the previous sub-steps in Step 6 to definitions offered by Guyatt et al. in Table 1 [18]. It should be noted that the steps in this component are a significant departure from the guidelines from Mokkink et al. [10]. (Mokkink et al. mostly focused their GRADE guidelines on Box 1 (EBP instrument development) and Box 2 (content validity). Our guidelines attempt to consider all Boxes (i.e.,1–10) and their ROB assessment, albeit in numerical values, that can be summarized based on the values from each box. Additionally, the process that Mokkink et al. outlined requires a lot of time extracting psychometric data and rating of those psychometric measurement properties. However, Mokkink et al. do not use those data in Step 7, but we have decided it is important to factor that into this final step [10]. As a result, we will use the guidelines below to assign a methodological quality standard or label based on the definitions, explanations, and guidelines in Table 1.

8) **Comprehensive reporting**

This protocol has been developed based on the updated COSMIN guideline for systematic reviews of PROMs including use of the COSMIN Risk of Bias checklist and criteria for good measurement properties [10]. The completed review will be reported according to the PRISMA-COSMIN for outcome measurement instruments (OMIs) 2024 reporting guideline [19], in line with PRISMA 2020 extensions for systematic reviews of outcome measurement instruments. We will aim to publish the full systematic review in PLOS One.

## Supporting information

S1 Checklist EBP measure Instr PRISMA-P SystRev checklist. (DOCX)

S1 Appendix Appendix A: The systematic review protocol registration in PROSPERO under the identifier CRD42024564908. (PDF)

S2 Appendix Appendix B: A complete list of inclusion and exclusion criteria for the proposed systematic review. (DOCX)

S3 Appendix Appendix C: Systematic review search strategy for each database. (DOCX)

S4 Appendix Appendix D: Systematic review data extraction methods. (XLSX)

S5 Appendix Appendix E: A template to evaluate the quality of the EBP competence measurement instruments for steps 6.1, 6.2, and 6.3. (XLSX)

S6 Appendix Appendix F: A template to summarize the results of the systematic review including steps 6.4, 6.5, and 6.6. (XLSX)

## References

[1] Albarqouni L, Hoffmann T, Straus S, Olsen NR, Young T, Ilic D, et al. Core competencies in evidence-based practice for health professionals: consensus statement based on a systematic review and Delphi survey. JAMA Netw Open. 2018;1(2):e180281. doi: 10.1001/jamanetworkopen.2018.0281 30646073

[2] Lehane E, Leahy-Warren P, O’Riordan C, Savage E, Drennan J, O’Tuathaigh C, et al. Evidence-based practice education for healthcare professions: an expert view. BMJ Evid Based Med. 2019;24(3):103–8. doi: 10.1136/bmjebm-2018-111019 30442711 PMC6582731

[3] Dawes M, Summerskill W, Glasziou P, Cartabellotta A, Martin J, Hopayian K, et al. Sicily statement on evidence-based practice. BMC Med Educ. 2005;5(1):1. doi: 10.1186/1472-6920-5-1 15634359 PMC544887

[4] Tilson JK, Kaplan SL, Harris JL, Hutchinson A, Ilic D, Niederman R, et al. Sicily statement on classification and development of evidence-based practice learning assessment tools. BMC Med Educ. 2011;11:78. doi: 10.1186/1472-6920-11-78 21970731 PMC3221624

[5] Bloom B, Engelhart M, Furst E, Hill W, Krathwohl D. Taxonomy of educational objectives: the classification of educational goals. Handbook 1: cognitive domain. New York: David McKay; 1956.

[6] Mills J-A, Middleton JW, Schafer A, Fitzpatrick S, Short S, Cieza A. Proposing a re-conceptualisation of competency framework terminology for health: a scoping review. Hum Resour Health. 2020;18(1):15. doi: 10.1186/s12960-019-0443-8 32085739 PMC7035756

[7] Van Der Vleuten CPM. Competency-based education is beneficial for professional development. Perspect Med Educ. 2015;4(6):323–5. doi: 10.1007/S40037-015-0232-6 26553242 PMC4673061

[8] Shaneyfelt T, Baum KD, Bell D, Feldstein D, Houston TK, Kaatz S, et al. Instruments for evaluating education in evidence-based practice: a systematic review. JAMA. 2006;296(9):1116–27. doi: 10.1001/jama.296.9.1116 16954491

[9] Landsverk NG, Olsen NR, Brovold T. Instruments measuring evidence-based practice behavior, attitudes, and self-efficacy among healthcare professionals: a systematic review of measurement properties. Implement Sci. 2023;18(1):42. doi: 10.1186/s13012-023-01301-3 37705031 PMC10500884

[10] Mokkink LB, Elsman EBM, Terwee CB. COSMIN guideline for systematic reviews of patient-reported outcome measures version 2.0. Qual Life Res. 2024;33(11):2929–39. doi: 10.1007/s11136-024-03761-6 39198348 PMC11541334

[11] Haavisto E, Siltanen H, Tolvanen A, Holopainen A. Instruments for assessing healthcare professionals’ knowledge and skills of evidence-based practice: a scoping review. J Clin Nurs. 2023;32(15–16):4391–407. doi: 10.1111/jocn.16506 36229896

[12] Page MJ, McKenzie JE, Bossuyt PM, Boutron I, Hoffmann TC, Mulrow CD, et al. The PRISMA 2020 statement: an updated guideline for reporting systematic reviews. BMJ. 2021;n71. doi: 10.1136/bmj.n71 PMC8005924 33782057

[13] Belita E, Squires JE, Yost J, Ganann R, Burnett T, Dobbins M. Measures of evidence-informed decision-making competence attributes: a psychometric systematic review. BMC Nurs. 2020;19:44. doi: 10.1186/s12912-020-00436-8 32514242 PMC7254762

[14] Roberge-Dao J, Maggio LA, Zaccagnini M, Rochette A, Shikako-Thomas K, Boruff J, et al. Quality, methods, and recommendations of systematic reviews on measures of evidence-based practice: an umbrella review. JBI Evid Synth. 2022;20(4):1004–73. doi: 10.11124/JBIES-21-00118 35220381

[15] Ruzafa-Martinez M, Lopez-Iborra L, Moreno-Casbas T, Madrigal-Torres M. Development and validation of the competence in evidence based practice questionnaire (EBP-COQ) among nursing students. BMC Med Educ. 2013;13:19. doi: 10.1186/1472-6920-13-19 23391040 PMC3598337

[16] Upton D, Upton P, Scurlock-Evans L. The reach, transferability, and impact of the Evidence-Based Practice Questionnaire: a methodological and narrative literature review. Worldviews Evid Based Nurs. 2014;11(1):46–54. doi: 10.1111/wvn.12019 24447383

[17] Perez JL, Mosher ZA, Watson SL, Sheppard ED, Brabston EW, McGwin G Jr, et al. Readability of orthopaedic patient-reported outcome measures: is there a fundamental failure to communicate? Clin Orthop Relat Res. 2017;475(8):1936–47. doi: 10.1007/s11999-017-5339-0 28374349 PMC5498383

[18] Guyatt GH, Oxman AD, Vist GE, Kunz R, Falck-Ytter Y, Alonso-Coello P, et al. GRADE: an emerging consensus on rating quality of evidence and strength of recommendations. BMJ. 2008;336(7650):924–6. doi: 10.1136/bmj.39489.470347.AD 18436948 PMC2335261

[19] Elsman EBM, Mokkink LB, Terwee CB, Beaton D, Gagnier JJ, Tricco AC, et al. Guideline for reporting systematic reviews of outcome measurement instruments (OMIs): PRISMA-COSMIN for OMIs 2024. J Clin Epidemiol. 2024;173:111422. doi: 10.1016/j.jclinepi.2024.111422 38849061

