# Supplementary material for: Instruments that measure evidence-based practice knowledge, skills, and attitudes among health professions students: A systematic review protocol
Source: PLoS One. 2026 Jul 13;21(7):e0347078. doi: 10.1371/journal.pone.0347078 (PMC13362092; doi:10.1371/journal.pone.0347078)
Supplement: S1 Appendix — (PDF) [file pone.0347078.s002.pdf]

NIHR | National Institute for Health and Care Research

PROSPERO
International prospective register of systematic reviews

Instruments that measure evidence-based practice knowledge, confidence, attitudes, and practice among health professions' students: A systematic review

Daniil Stolear, Lynne Lafave, Breda Eubank, Jenelle McAllister, Mark Lafave

To enable PROSPERO to focus on COVID-19 submissions, this registration record has undergone basic automated checks for eligibility and is published exactly as submitted. PROSPERO has never provided peer review, and usual checking by the PROSPERO team does not endorse content. Therefore, automatically published records should be treated as any other PROSPERO registration. Further detail is provided here.

Citation
Daniil Stolear, Lynne Lafave, Breda Eubank, Jenelle McAllister, Mark Lafave. Instruments that measure evidence-based practice knowledge, confidence, attitudes, and practice among health professions' students: A systematic review. PROSPERO 2024 Available from https://www.crd.york.ac.uk/PROSPERO/view/CRD42024564908

REVIEW TITLE AND BASIC DETAILS

Review title
Instruments that measure evidence-based practice knowledge, confidence, attitudes, and practice among health professions' students: A systematic review

Review objectives
What is the psychometric quality and measurement properties utilized in instruments that measure EBP knowledge, confidence, attitudes, and/or practice concepts for students within the context of post-secondary education?

Keywords
Evidence based practice, health disciplines, health fields, questionnaires, tools

SEARCHING AND SCREENING

Searches

MEDLINE, CINAHL, SPORTDiscus, and PsycINFO all via EBSCO. Preliminary searches were carried out June 28-July 1, 2024. Only articles in English will be included however we will not limit this in the search strategy; these studies will be filtered out during title/abstract screening.

Study design

This systematic review will consider both experimental and quasi-experimental study designs including randomized controlled trials, non-randomized controlled trials, before and after studies and interrupted time-series studies. In addition, analytical observational studies including prospective and retrospective cohort studies. Qualitative studies will also be considered that focus on qualitative data from students on instruments measuring EBP related outcomes. These can be in relation to attitudes, self-efficacy, or reaction to their EBP educational experience (Tilson et al., 2011). Scoping, systematic, and umbrella reviews as well as meta-analyses will be included. Dissertations, editorials, theses, conference abstracts, and theoretical papers will be excluded.

ELIGIBILITY CRITERIA

Condition or domain being studied

Evidence-based practice (EBP) in health professions

Population

Students enrolled in post-secondary studies within health fields that utilize EBP at an institution. Disciplines outside of health fields that utilize EBP will be excluded, such as but not limited to primary school teachers.

Intervention(s) or exposure(s)

Instruments that measure at least one of benefit to patients, behaviors, skills, knowledge, self-efficacy, attitudes, or reaction to educational experience. Studies must report psychometric properties of such instruments; studies that do not report on psychometric properties will be excluded.

Comparator(s) or control(s)

No comparisons between instruments will be made

Context

Post-secondary institutions and health professions education settings.

OUTCOMES TO BE ANALYSED

Main outcomes

Extracting the domains of EBP (Tilson et al., 2011) captured by instruments measuring them in student populations as well as the presence of the psychometric properties outlined by COSMIN (Mokkink et al., 2018) within such instruments.

Measures of effect

EBP domains: benefit to patients, behaviors, skills, knowledge, self-efficacy, attitudes, reaction to the educational experience (Tilson et al., 2011). Psychometric properties: content validity, structural validity, construct validity, internal consistency, reliability, measurement error, translation, and responsiveness (Mokkink et al., 2018).

Additional outcomes

Not applicable

DATA COLLECTION PROCESS

Data extraction (selection and coding)

For inclusion, articles must: be peer-reviewed journal articles, contain an instrument evaluating EBP, contain sufficient description of the EBP measure to allow for analysis, contain quantitative results of either administering the instrument or development of instrument, measure at least one EBP domain (benefit to patients, behaviors, skills, knowledge, self-efficacy, attitudes, or reaction to the educational experience), identify and report psychometric properties of the instrument(s), and have students as part of the sample that the instrument was administered to. Studies with an exclusive focus on measures of research utilization, peripheral evidence-based practice concepts, or effects of implementation strategies will be excluded. Dissertations, editorials, theses, conference abstracts, theoretical papers, or studies that are not available in English will also be excluded. Studies will undergo title/abstract screening to assess for relevancy and exclusion criteria. Studies that are not excluded will proceed to full-text screening against the inclusion criteria. The data from the studies not excluded after full-text screening will be extracted by two or more reviewers blinded to each other into a spreadsheet. Descriptive extraction data consists of: Instrument name, whether it has been validated previously (yes/no), student sample size, discipline of students, and their field. EBP outcome extraction data (whether instrument measures the given outcomes) consists of: benefit to patients, behaviors, skills, knowledge, self-efficacy, attitudes, reaction to the educational experience (all yes/no). Psychometric property extraction data consists of: PROM development, content validity, structural validity, internal consistency, cross-cultural validity, reliability, measurement error, criterion validity, hypotheses testing for construct validity, and responsiveness, categorized as one of very good, adequate, doubtful, inadequate, or not applicable based on the COSMIN guidelines (Mokkink et al., 2018).

Risk of bias (quality) assessment

The instrument(s) used in each study will be evaluated for quality against the 10 COSMIN psychometric dimensions including PROM development, content validity, structural validity, internal consistency, cross-cultural validity, reliability, measurement error, criterion validity, hypotheses testing for construct validity, and responsiveness (Mokkink et al., 2018). They will be categorized into quality categories of very good, adequate, doubtful, inadequate, or not applicable in each category.

PLANNED DATA SYNTHESIS

Strategy for data synthesis

Descriptive data from the chosen studies will be presented visually through tables and charts, with a narrative summary provided for the relevance and potential limitations of the findings. The Risk of Bias assessment will be presented in the form of a table for the chosen studies. A PRISMA flow chart will present the process and quantitative data for study selection and exclusion.

Analysis of subgroups or subsets

Our review will report descriptive data (sample size, discipline, and field of work) on subgroups for studies that administer an instrument to more than one student group, but not any practicing professionals unless they are also students (e.g. practicum students). The quantitative scores or performance on the instruments administered to students and any subgroups are outside the scope of this systematic review and will not be extracted.

REVIEW AFFILIATION, FUNDING AND PEER REVIEW

Review team members

- Mr Daniil Stolear, Mount Royal University
- Dr Lynne Lafave, Mount Royal University
- Dr Breda Eubank, Mount Royal University
- Ms Jenelle McAllister, Mount Royal University
- Dr Mark Lafave, Mount Royal University

Review affiliation

Mount Royal University

Funding source

We received funding from the Health, Community, and Education Innovation Fund at Mount Royal University

Named contact

Daniil Stolear. 4825 Mt Royal Gate SW, Calgary, AB T3E 6K6
dstol091@mtroyal.ca

TIMELINE OF THE REVIEW

Review timeline

Start date: 10 July 2024. End date: 31 August 2024

Date of first submission to PROSPERO

09 July 2024

Date of registration in PROSPERO

20 July 2024

CURRENT REVIEW STAGE

Publication of review results
The intention is to publish the review once completed.The review will be published in English

Stage of the review at this submission

Review stage	Started	Completed
Pilot work		
Formal searching/study identification		
Screening search results against inclusion criteria		
Data extraction or receipt of IP		
Risk of bias/quality assessment		
Data synthesis		

Review status
The review is currently planned or ongoing.

ADDITIONAL INFORMATION

Additional information

Collaborators

- **Dr Colin King**, Acadia University
- **Dr Loriann Hynes**, York University

PROSPERO version history

- Version 1.1 published on 20 Jul 2024
- Version 1.0 published on 20 Jul 2024

Review conflict of interest
None known

Country
Canada

Medical Subject Headings
Evidence-Based Practice; Humans; Mental Processes; Psychometrics; Students, Health Occupations

Disclaimer
The content of this record displays the information provided by the review team. PROSPERO does not peer review registration records or endorse their content.

PROSPERO accepts and posts the information provided in good faith; responsibility for record content rests with the review team. The owner of this record has affirmed that the information

provided is truthful and that they understand that deliberate provision of inaccurate information may be construed as scientific misconduct.

PROSPERO does not accept any liability for the content provided in this record or for its use. Readers use the information provided in this record at their own risk.

Any enquiries about the record should be referred to the named review contact
