# Supplementary material for: Instruments that measure evidence-based practice knowledge, skills, and attitudes among health professions students: A systematic review protocol
Source: PLoS One. 2026 Jul 13;21(7):e0347078. doi: 10.1371/journal.pone.0347078 (PMC13362092; doi:10.1371/journal.pone.0347078)
Supplement: S2 Appendix — (DOCX) [file pone.0347078.s003.docx]

**Inclusion Criteria:**

- Peer-reviewed journal articles
- Contains a self-reported instrument(s) that measures at least one of the following dimensions pertaining to evidence-based practice: attitude (including self-efficacy, beliefs, and motivation), or practice/intention to practice (including behaviours, competency, and patient benefits)
- Contains sufficient description of instrument’s validation process
- Contains quantitative results of either administering instrument or development of instrument
- There is at least one health student* sample that the instrument was administered to
- Identifies and reports psychometric properties of EBP measure(s)

**Exclusion Criteria:**

- Dissertations, reviews, protocols, editorials, theses, conference abstracts, erratums, theoretical papers, or exclusively qualitative research
- The instrument measures knowledge and/or skills exclusively
- ~~Not available in English~~
- No health student* sample
- Does not report or discuss specific EBP measures and psychometric properties (eg, papers that say ‘Questionnaire was developed and administered’ and carry no discussion of the instrument and its properties)
- The instrument measures student perceptions/feedback for the EBP intervention exclusively (eg, no knowledge, skills, or attitudes, just what they thought of the new EBP curriculum/course)
- The instrument is not self-reported by the student sample
- Examines measures of “research utilization,” as this is conceptually distinct from EBP (although often used interchangeably), and the focus of our review is on the broader aggregate process of EBP (Roberge-Dao et al., 2022)
- Examines measures assessing more peripheral concepts to EBP (eg, critical thinking, clinical reasoning or was only for continuing education purposes)
- Examines measures for a specific area of clinical practice only. For example, like stroke or high blood pressure.
- Focuses on the effect of implementation strategies exclusively (for example, is focused on an intervention strategy like a teaching technique or mentoring program.

*Health students are defined as students or trainees currently pursuing studies or training for a profession which involves providing treatment/assistance/counselling to human patients or clients. Professionals enrolled in continuing education courses are NOT part of this definition (i.e. physicians taking a continuing education course in EBP).
