# Supplementary material for: Instruments that measure evidence-based practice knowledge, skills, and attitudes among health professions students: A systematic review protocol
Source: PLoS One. 2026 Jul 13;21(7):e0347078. doi: 10.1371/journal.pone.0347078 (PMC13362092; doi:10.1371/journal.pone.0347078)
Supplement: S3 Appendix — (DOCX) [file pone.0347078.s004.docx]

**SEARCH STRATEGY**

Databases included in our search: MEDLINE, CINAHL, SPORTDiscus, PsycINFO

| **MEDLINE** |  |
| --- | --- |
| Concept 1: health professional students | (MH "Students, Health Occupations+")  OR  AB ((health OR healthcare OR “health care” OR "health-care" OR nurs* OR medical OR doctor* OR physician* OR pharmac* OR dentist* OR physiotherap* OR therap* OR “social work” OR "social worker" OR "social workers" OR paramed* OR dietetic* OR chiropract* OR acupunctur* OR nutrition* OR patholog* OR psychologis* OR midwi* OR counsello* OR "athletic trainer" OR "athletic training" OR "athletic trainers") N2 (student* OR trainee*)) OR TI ((health OR healthcare OR “health care” OR "health-care" OR nurs* OR medical OR doctor* OR physician* OR pharmac* OR dentist* OR physiotherap* OR therap* OR "social work" OR "social worker" OR "social workers" OR paramed* OR dietetic* OR chiropract* OR acupunctur* OR nutrition* OR patholog* OR psychologis* OR midwi* OR counsello* OR "athletic trainer" OR "athletic training" OR "athletic trainers") N2 (student* OR trainee*)) |
|  | **AND** |
| Concept 2: evidence based practice | (MH "Evidence-Based Practice+")  OR  AB (("evidence based" OR "evidence-based" OR "evidence informed" OR "evidence-informed" OR "research based" OR "research-based" OR "knowledge based" OR "knowledge-based")) N2 (practice* OR healthcare OR "health care" OR "health-care" OR "decision-making" OR "decision making" OR treatment* OR medicine OR nursing)) OR TI (("evidence based" OR "evidence-based" OR "evidence informed" OR "evidence-informed" OR "research based" OR "research-based" OR "knowledge based" OR "knowledge-based")) N2 (practice* OR healthcare OR "health care" OR "health-care" OR "decision-making" OR "decision making" OR treatment* OR medicine OR nursing))  OR  AB (EBP OR EIDM OR EBN OR EMB OR EIP) OR TI (EBP OR EIDM OR EBN OR EBM OR EIP) |
|  | **AND** |
| Concept 3: survey instruments | (MH "Surveys and Questionnaires+") OR (MH "Psychological Tests+") OR (MH "Reproducibility of Results+") OR (MH "Psychometrics") OR (MH "Social Validity, Research")  OR  AB (instrument* OR scale* OR tool OR tools OR assess* OR scoring* OR score* OR survey* OR questionnaire* OR measur* OR valid* OR psychometric* OR "content validity" OR "structural validity" OR "construct validity" OR "internal consistency" OR reliab* OR "measurement error" OR "translation") OR TI (instrument* OR scale* OR tool OR tools OR assess* OR scoring* OR score* OR survey* OR questionnaire* OR measur* OR valid* OR psychometric* OR "content validity" OR "structural validity" OR "construct validity" OR "internal consistency" OR reliab* OR "measurement error" OR "translation") |
|  | **AND** |
| Concept 4: knowledge, attitude, competence | (MH "Attitude of Health Personnel") OR (MH "Health Knowledge, Attitudes, Practice") OR (MH "Professional Competence+") OR  (MH "Self Concept") OR (MH "Self Efficacy") OR (MH "Self-Assessment") OR (MH "Behavior+")  OR  AB (attitude* OR perception* OR opinion* OR belief* OR behavior* OR behaviour* OR competen* OR "self-esteem" OR "self esteem" OR "self-belief" OR "self belief" OR "self-concept" OR "self concept" OR knowledge OR perform* OR skill* OR confiden* OR "practice") OR TI (attitude* OR perception* OR opinion* OR belief* OR behavior* OR behaviour* OR competen* OR "self-esteem" OR "self esteem" OR "self-belief" OR "self belief" OR "self-concept" OR "self concept" OR knowledge OR perform* OR skill* OR confiden* OR "practice") |

| **CINAHL** |  |
| --- | --- |
| Concept 1: health professional students | (MH "Students, Health Occupations+")  OR  AB ((health OR healthcare OR "health care" OR "health-care" OR nurs* OR medical OR doctor* OR physician* OR pharmac* OR dentist* OR physiotherap* OR therap* OR "social work" OR "social worker" OR "social workers" OR paramed* OR dietetic* OR chiropract* OR acupunctur* OR nutrition* OR patholog* OR psychologis* OR midwi* OR counsello* OR "athletic trainer" OR "athletic training" OR "athletic trainers") N2 (student* OR trainee*)) OR TI ((health OR healthcare OR “health care” OR "health-care" OR nurs* OR medical OR doctor* OR physician* OR pharmac* OR dentist* OR physiotherap* OR therap* OR "social work" OR "social worker" OR "social workers" OR paramed* OR dietetic* OR chiropract* OR acupunctur* OR nutrition* OR patholog* OR psychologis* OR midwi* OR counsello* OR "athletic trainer" OR "athletic training" OR "athletic trainers") N2 (student* OR trainee*)) |
|  | **AND** |
| Concept 2: evidence based practice | (MH "Professional Practice, Evidence-Based+")  OR  AB (("evidence based" OR "evidence-based" OR "evidence informed" OR "evidence-informed" OR "research based" OR "research-based" OR "knowledge based" OR "knowledge-based")) N2 (practice* OR healthcare OR "health care" OR "health-care" OR "decision-making" OR "decision making" OR treatment* OR medicine OR nursing)) OR TI (("evidence based" OR "evidence-based" OR "evidence informed" OR "evidence-informed" OR "research based" OR "research-based" OR "knowledge based" OR "knowledge-based")) N2 (practice* OR healthcare OR "health care" OR "health-care" OR "decision-making" OR "decision making" OR treatment* OR medicine OR nursing))  OR  AB (EBP OR EIDM OR EBN OR EMB OR EIP) OR TI (EBP OR EIDM OR EBN OR EBM OR EIP) |
|  | **AND** |
| Concept 3: survey instruments | (MH "Surveys") OR (MH "Questionnaires+") OR (MH "Psychological Tests+") OR (MH "Reproducibility of Results") OR (MH "Psychometrics") OR (MH "Validation Studies")  OR  AB (instrument* OR scale* OR tool OR tools OR assess* OR scoring* OR score* OR survey* OR questionnaire* OR measur* OR valid* OR psychometric* OR "content validity" OR "structural validity" OR "construct validity" OR "internal consistency" OR reliab* OR "measurement error" OR "translation") OR TI (instrument* OR scale* OR tool OR tools OR assess* OR scoring* OR score* OR survey* OR questionnaire* OR measur* OR valid* OR psychometric* OR "content validity" OR "structural validity" OR "construct validity" OR "internal consistency" OR reliab* OR "measurement error" OR "translation") |
|  | **AND** |
| Concept 4: knowledge, attitude, competence | (MH "Attitude of Health Personnel+") OR (MH "Student Attitudes+") OR (MH "Knowledge+") OR (MH "Professional Competence") OR (MH "Clinical Competence+") OR (MH "Self Concept") OR (MH "Confidence") OR (MH "Self-Efficacy") OR (MH "Self Assessment") OR (MH "Behavior+")  OR  AB (attitude* OR perception* OR opinion* OR belief* OR behavior* OR behaviour* OR competen* OR "self-esteem" OR "self esteem" OR "self-belief" OR "self belief" OR "self-concept" OR "self concept" OR knowledge OR perform* OR skill* OR confiden* OR "practice") OR TI (attitude* OR perception* OR opinion* OR belief* OR behavior* OR behaviour* OR competen* OR "self-esteem" OR "self esteem" OR "self-belief" OR "self belief" OR "self-concept" OR "self concept" OR knowledge OR perform* OR skill* OR confiden* OR "practice") |

| **SPORTDiscus** |  |
| --- | --- |
| Concept 1: health professional students | (DE "STUDENTS") OR (DE "MEDICAL students") OR (DE "PHYSICAL therapy students")  OR  AB ((health OR healthcare OR “health care” OR "health-care" OR nurs* OR medical OR doctor* OR physician* OR pharmac* OR dentist* OR physiotherap* OR therap* OR “social work” OR "social worker" OR "social workers" OR paramed* OR dietetic* OR chiropract* OR acupunctur* OR nutrition* OR patholog* OR psychologis* OR midwi* OR counsello* OR "athletic trainer" OR "athletic training" OR "athletic trainers") N2 (student* OR trainee*)) OR TI ((health OR healthcare OR “health care” OR "health-care" OR nurs* OR medical OR doctor* OR physician* OR pharmac* OR dentist* OR physiotherap* OR therap* OR "social work" OR "social worker" OR "social workers" OR paramed* OR dietetic* OR chiropract* OR acupunctur* OR nutrition* OR patholog* OR psychologis* OR midwi* OR counsello* OR "athletic trainer" OR "athletic training" OR "athletic trainers") N2 (student* OR trainee*)) |
|  | **AND** |
| Concept 2: evidence based practice | DE "EVIDENCE-based medicine"  OR  AB (("evidence based" OR "evidence-based" OR "evidence informed" OR "evidence-informed" OR "research based" OR "research-based" OR "knowledge based" OR "knowledge-based")) N2 (practice* OR healthcare OR "health care" OR "health-care" OR "decision-making" OR "decision making" OR treatment* OR medicine OR nursing)) OR TI (("evidence based" OR "evidence-based" OR "evidence informed" OR "evidence-informed" OR "research based" OR "research-based" OR "knowledge based" OR "knowledge-based")) N2 (practice* OR healthcare OR "health care" OR "health-care" OR "decision-making" OR "decision making" OR treatment* OR medicine OR nursing))  OR  AB (EBP OR EIDM OR EBN OR EMB OR EIP) OR TI (EBP OR EIDM OR EBN OR EBM OR EIP) |
|  | **AND** |
| Concept 3: survey instruments | DE "PSYCHOLOGICAL tests"  OR  AB (instrument* OR scale* OR tool OR tools OR assess* OR scoring* OR score* OR survey* OR questionnaire* OR measur* OR valid* OR psychometric* OR "content validity" OR "structural validity" OR "construct validity" OR "internal consistency" OR reliab* OR "measurement error" OR "translation") OR TI (instrument* OR scale* OR tool OR tools OR assess* OR scoring* OR score* OR survey* OR questionnaire* OR measur* OR valid* OR psychometric* OR "content validity" OR "structural validity" OR "construct validity" OR "internal consistency" OR reliab* OR "measurement error" OR "translation") |
|  | **AND** |
| Concept 4: knowledge, attitude, competence | (DE "CONFIDENCE") OR (DE "STUDENT attitudes") OR (DE "COLLEGE student attitudes") OR (DE "SELF-efficacy")  OR  (DE "SELF-perception") OR (DE "SELF-evaluation")  OR  AB (attitude* OR perception* OR opinion* OR belief* OR behavior* OR behaviour* OR competen* OR "self-esteem" OR "self esteem" OR "self-belief" OR "self belief" OR "self-concept" OR "self concept" OR knowledge OR perform* OR skill* OR confiden* OR "practice") OR TI (attitude* OR perception* OR opinion* OR belief* OR behavior* OR behaviour* OR competen* OR "self-esteem" OR "self esteem" OR "self-belief" OR "self belief" OR "self-concept" OR "self concept" OR knowledge OR perform* OR skill* OR confiden* OR "practice") |

| **PsycINFO** |  |
| --- | --- |
| Concept 1: health professional students | (DE "Postgraduate Students") OR (DE "Graduate Students") OR (DE "Dental Students") OR (DE "Medical Students")  OR  (DE "Nursing Students") OR (DE "Therapist Trainees") OR (DE "Counselor Trainees")  OR  AB ((health OR healthcare OR “health care” OR "health-care" OR nurs* OR medical OR doctor* OR physician* OR pharmac* OR dentist* OR physiotherap* OR therap* OR “social work” OR "social worker" OR "social workers" OR paramed* OR dietetic* OR chiropract* OR acupunctur* OR nutrition* OR patholog* OR psychologis* OR midwi* OR counsello* OR "athletic trainer" OR "athletic training" OR "athletic trainers") N2 (student* OR trainee* OR "in training")) OR TI ((health OR healthcare OR “health care” OR "health-care" OR nurs* OR medical OR doctor* OR physician* OR pharmac* OR dentist* OR physiotherap* OR therap* OR "social work" OR "social worker" OR "social workers" OR paramed* OR dietetic* OR chiropract* OR acupunctur* OR nutrition* OR patholog* OR psychologis* OR midwi* OR counsello* OR "athletic trainer" OR "athletic training" OR "athletic trainers") N2 (student* OR trainee* OR "in training")) |
|  | **AND** |
| Concept 2: evidence based practice | DE "Evidence Based Practice"  OR  AB (("evidence based" OR "evidence-based" OR "evidence informed" OR "evidence-informed" OR "research based" OR "research-based" OR "knowledge based" OR "knowledge-based")) N2 (practice* OR healthcare OR "health care" OR "health-care" OR "decision-making" OR "decision making" OR treatment* OR medicine OR nursing)) OR TI (("evidence based" OR "evidence-based" OR "evidence informed" OR "evidence-informed" OR "research based" OR "research-based" OR "knowledge based" OR "knowledge-based")) N2 (practice* OR healthcare OR "health care" OR "health-care" OR "decision-making" OR "decision making" OR treatment* OR medicine OR nursing))  OR  AB (EBP OR EIDM OR EBN OR EMB OR EIP) OR TI (EBP OR EIDM OR EBN OR EBM OR EIP) |
|  | **AND** |
| Concept 3: survey instruments | (DE "Surveys") OR (DE "Online Surveys") OR (DE "Questionnaires") OR (DE "Professional Measures") OR (DE "Health Care Personnel Measures") OR (DE "Mental Health Care Personnel Measures") OR (DE "Testing") OR (DE "Psychometrics") OR (DE "Attitude Measures")  OR  AB (instrument* OR scale* OR tool OR tools OR assess* OR scoring* OR score* OR survey* OR questionnaire* OR measur* OR valid* OR psychometric* OR "content validity" OR "structural validity" OR "construct validity" OR "internal consistency" OR reliab* OR "measurement error" OR "translation") OR TI (instrument* OR scale* OR tool OR tools OR assess* OR scoring* OR score* OR survey* OR questionnaire* OR measur* OR valid* OR psychometric* OR "content validity" OR "structural validity" OR "construct validity" OR "internal consistency" OR reliab* OR "measurement error" OR "translation") |
|  | **AND** |
| Concept 4: knowledge, attitude, competence | (DE "Knowledge (General)") OR (DE "Attitudes") OR (DE "Student Attitudes") OR (DE "Health Personnel Attitudes") OR (DE "Caregiver Attitudes") OR (DE "Counselor Attitudes") OR (DE "Psychologist Attitudes") OR (DE "Therapist Attitudes") OR (DE "Self-Efficacy") OR (DE "Self-Perception") OR (DE "Self-Concept") OR (DE "Self-Confidence") OR (DE "Competence") OR (DE "Ability") OR (DE "Professional Competence") OR (DE "Behavior")  OR  AB (attitude* OR perception* OR opinion* OR belief* OR behavior* OR behaviour* OR competen* OR "self-esteem" OR "self esteem" OR "self-belief" OR "self belief" OR "self-concept" OR "self concept" OR knowledge OR perform* OR skill* OR confiden* OR "practice") OR TI (attitude* OR perception* OR opinion* OR belief* OR behavior* OR behaviour* OR competen* OR "self-esteem" OR "self esteem" OR "self-belief" OR "self belief" OR "self-concept" OR "self concept" OR knowledge OR perform* OR skill* OR confiden* OR "practice") |
