# Supplementary material for: Instruments that measure evidence-based practice knowledge, skills, and attitudes among health professions students: A systematic review protocol
Source: PLoS One. 2026 Jul 13;21(7):e0347078. doi: 10.1371/journal.pone.0347078 (PMC13362092; doi:10.1371/journal.pone.0347078)
Supplement: Attachment — Submitted filename: Response to Reviewers.June.10.2026.docx [file pone.0347078.s008.docx]

**Response to Reviewers**

PONE-D-26-14849

Instruments that measure evidence-based practice knowledge, skills, and attitudes among health professions students: A systematic review protocol

PLOS One

Dear Dr. Lafave,

Thank you for submitting your manuscript to PLOS ONE. After careful consideration, we feel that it has merit but does not fully meet PLOS ONE’s publication criteria as it currently stands. Therefore, we invite you to submit a revised version of the manuscript that addresses the points raised during the review process.

**Thank you for the review and suggestions. We have included a response to the editorial team and reviewers in this “response to reviewers” file. We have copied and pasted the comments/suggestions and included them in this file and our responses are directly below and bolded.**

==============================
**ACADEMIC EDITOR:**

- The authors are requested to focus on the reviewer's comments and follow up as either
  (a) Provide an explicit and scientifically defensible rationale for how GRADE criteria were operationalized in the item development process, with reference to supporting methodological literature; or

**We have addressed this request in the reviewer comments below.**

(b) Revise the methods section to accurately reflect the instrument development framework employed

**We have addressed this request in the reviewer comments below.**

==============================

Please submit your revised manuscript by Jul 24 2026 11:59PM. If you will need more time than this to complete your revisions, please reply to this message or contact the journal office at [plosone@plos.org](mailto:plosone@plos.org). When you're ready to submit your revision, log on to <https://www.editorialmanager.com/pone/> and select the 'Submissions Needing Revision' folder to locate your manuscript file.

Please include the following items when submitting your revised manuscript:

- A letter that responds to each point raised by the academic editor and reviewer(s). You should upload this letter as a separate file labeled 'Response to Reviewers'.

**Done.**

- A marked-up copy of your manuscript that highlights changes made to the original version. You should upload this as a separate file labeled 'Revised Manuscript with Track Changes'.

**Done. We tracked changes by highlighting those changes in BOLD and YELLOW. We made the stylistic changes, but did not use any track changes, rather we just made the adjustments to font sizes and removed the previous boxes. This should follow all the heading requirements that are required.**

- An unmarked version of your revised paper without tracked changes. You should upload this as a separate file labeled 'Manuscript'.

**Done.**

If you would like to make changes to your financial disclosure, please include your updated statement in your cover letter. Guidelines for resubmitting your figure files are available below the reviewer comments at the end of this letter.

**We included our financial disclosures originally and it remains accurate.**

If applicable, we recommend that you deposit your laboratory protocols in [protocols.io](http://protocols.io/) to enhance the reproducibility of your results. Protocols.io assigns your protocol its own identifier (DOI) so that it can be cited independently in the future. For instructions see: [https://journals.plos.org/plosone/s/submission-guidelines#loc-laboratory-protocols](https://track.editorialmanager.com/CL0/https:%2F%2Fjournals.plos.org%2Fplosone%2Fs%2Fsubmission-guidelines%23loc-laboratory-protocols/1/010f019eaf74f431-9bf2e61a-ce6b-47c6-949d-0c716dfc1c8b-000000/MaXmCYCnVjWu_2n-ser8GRinylDIPcEPtckvOvnDTiE=258). Additionally, PLOS ONE offers an option for publishing peer-reviewed Lab Protocol articles, which describe protocols hosted on [protocols.io](http://protocols.io/). Read more information on sharing protocols at [https://plos.org/protocols?utm_medium=editorial-email&utm_source=authorletters&utm_campaign=protocols](https://track.editorialmanager.com/CL0/https:%2F%2Fplos.org%2Fprotocols%3Futm_medium=editorial-email%26utm_source=authorletters%26utm_campaign=protocols/1/010f019eaf74f431-9bf2e61a-ce6b-47c6-949d-0c716dfc1c8b-000000/Y2EkTJGhQ8FPMbaZ72zGJJpyGLEaMtINfprUrxwWQGI=258).

**This is not applicable.**

As the corresponding author, your ORCID iD is verified in the submission system and will appear in the published article. PLOS supports the use of ORCID, and we encourage all coauthors to register for an ORCID iD and use it as well. Please encourage your coauthors to verify their ORCID iD within the submission system before final acceptance, as unverified ORCID iDs will not appear in the published article. *Only* the individual author can complete the verification step; PLOS staff *cannot* verify ORCID iDs on behalf of authors.

**We have included ORCID for authors who have it.**

Journal Requirements:
When submitting your revision, we need you to address these additional requirements.
1. Please ensure that your manuscript meets PLOS ONE's style requirements, including those for file naming. The PLOS ONE style templates can be found at
[https://journals.plos.org/plosone/s/file?id=wjVg/PLOSOne_formatting_sample_main_body.pdf](https://track.editorialmanager.com/CL0/https:%2F%2Fjournals.plos.org%2Fplosone%2Fs%2Ffile%3Fid=wjVg%2FPLOSOne_formatting_sample_main_body.pdf/1/010f019eaf74f431-9bf2e61a-ce6b-47c6-949d-0c716dfc1c8b-000000/cF8lv3wedAwfsRT32VVrfd68qXgUfs3HL-BzQt1Lt04=258) and [https://journals.plos.org/plosone/s/file?id=ba62/PLOSOne_formatting_sample_title_authors_affiliations.pdf](https://track.editorialmanager.com/CL0/https:%2F%2Fjournals.plos.org%2Fplosone%2Fs%2Ffile%3Fid=ba62%2FPLOSOne_formatting_sample_title_authors_affiliations.pdf/1/010f019eaf74f431-9bf2e61a-ce6b-47c6-949d-0c716dfc1c8b-000000/aBP_l_OLHPw4x8rNucEJGADQe0Eu2EcOV1qRzhh82J8=258)

**We have edited the manuscript to be compliant with PLOS ONE’s style requirements.**

2. Please provide a complete Data Availability Statement in the submission form, ensuring you include all necessary access information or a reason for why you are unable to make your data freely accessible. If your research concerns only data provided within your submission, please write "All data are in the manuscript and/or supporting information files" as your Data Availability Statement.

**We have added a data availability statement to the submission form as per your request.**

3. Please amend your authorship list in your manuscript file to include author Mark R. Lafave, Lynne Lafave, Breda Eubank.

**We have changed the author list accordingly.**

4. Please amend the manuscript submission data (via Edit Submission) to include author Lafave, MR., Lafave, LMZ, Eubank, B.,

**We have added the authors in the “edit submission” feature upon submission.**

5. Please include captions for your Supporting Information files at the end of your manuscript, and update any in-text citations to match accordingly. Please see our Supporting Information guidelines for more information: http://journals.plos.org/plosone/s/supporting-information.

**We have included captions for Appendices A through F at the bottom of the manuscript file on lines 551-561.**

Reviewers' comments:

Reviewer's Responses to Questions

**Comments to the Author**

1. Does the manuscript provide a valid rationale for the proposed study, with clearly identified and justified research questions?

The research question outlined is expected to address a valid academic problem or topic and contribute to the base of knowledge in the field.

Reviewer #1: Yes

Reviewer #2: Yes

2. Is the protocol technically sound and planned in a manner that will lead to a meaningful outcome and allow testing the stated hypotheses?

The manuscript should describe the methods in sufficient detail to prevent undisclosed flexibility in the experimental procedure or analysis pipeline, including sufficient outcome-neutral conditions (e.g. necessary controls, absence of floor or ceiling effects) to test the proposed hypotheses and a statistical power analysis where applicable. As there may be aspects of the methodology and analysis which can only be refined once the work is undertaken, authors should outline potential assumptions and explicitly describe what aspects of the proposed analyses, if any, are exploratory.

Reviewer #1: Partly

Reviewer #2: Yes

3. Is the methodology feasible and described in sufficient detail to allow the work to be replicable?


Descriptions of methods and materials in the protocol should be reported in sufficient detail for another researcher to reproduce all experiments and analyses. The protocol should describe the appropriate controls, sample size calculations, and replication needed to ensure that the data are robust and reproducible.

Reviewer #1: Yes

Reviewer #2: Yes

4. Have the authors described where all data underlying the findings will be made available when the study is complete?

The [PLOS Data policy](https://track.editorialmanager.com/CL0/https:%2F%2Fjournals.plos.org%2Fplosone%2Fs%2Fmaterials-and-software-sharing/1/010f019eaf74f431-9bf2e61a-ce6b-47c6-949d-0c716dfc1c8b-000000/nGMtjMKr1ZzRvx9w9sG_6eP0ySxGv9uZFWYwBsl0q9g=258) requires authors to make all data underlying the findings described in their manuscript fully available without restriction, with rare exception, at the time of publication. The data should be provided as part of the manuscript or its supporting information, or deposited to a public repository. For example, in addition to summary statistics, the data points behind means, medians and variance measures should be available. If there are restrictions on publicly sharing data—e.g. participant privacy or use of data from a third party—those must be specified.

Reviewer #1: Yes

Reviewer #2: Yes

5. Is the manuscript presented in an intelligible fashion and written in standard English?

PLOS ONE does not copyedit accepted manuscripts, so the language in submitted articles must be clear, correct, and unambiguous. Any typographical or grammatical errors should be corrected at revision, so please note any specific errors here.

Reviewer #1: Yes

Reviewer #2: Yes

6. Review Comments to the Author

Please use the space provided to explain your answers to the questions above and, if applicable, provide comments about issues authors must address before this protocol can be accepted for publication. You may also include additional comments for the author, including concerns about research or publication ethics.

You may also provide optional suggestions and comments to authors that they might find helpful in planning their study.


(Please upload your review as an attachment if it exceeds 20,000 characters)

Reviewer #1: This is a protocol for a structured review study, which has been termed a systematic review. I suggest adding:
a) The study question and the PICO strategy or its variations to be adopted.

**The PICO is already in the submitted manuscript on Lines 116-120.**

b) Which method will be used for primary studies? Observational or Clinical Trials? It is important to specify the method of the studies to be included to ensure the quality of the systematic review.

**The type of studies that are included in this systematic review are listed on Lines 170-175.**

c) The GRADE system is used to assess the quality of systematic review studies. Note why you will be working with primary studies to develop your review.

**Yes, we are including primary studies and more specifically, studies that measure one or multiple of the nine measurement properties. We have added lines 180-182 to clarify as per your suggestion.**

d) If observational primary studies are included, use the STROBE protocol; if clinical trials are included, use the Jadad Scale or even CONSORT – as the main instruments to measure the quality of the included studies.

**It is important to note that systematic review methodology for the development EBP measurement instruments or tools is unique relative to other clinical studies, observational studies, or randomized clinical trials. This is why we have followed a previously published article (Landsverk et al., 2018) that used the COSMIN guidelines for systematic reviews (Mokkink et al., 2024). Mokkink et al updated their protocol since Landsverk et al published their study in 2018, so we have used the updated version. STROBE, CONSORT and the Jadad Scale are all methods/protocols intended for a different type of study that we are proposing.**

Reviewer #2: It is a well written protocol. it would be nice to justify in the methods section why you have chosen this ROB method compared to other available ROB assessment tools.

**We agree that it is important to justify the methods we have chosen beyond following a previously published systematic review that employed the COSMIN guidelines for systematic reviews. We have added lines 131-134 and 139-141 to justify the methods we have chosen. Additionally, our current text on lines 150-160 offers where we have adjusted the methods, with rationale for them.**

7. PLOS authors have the option to publish the peer review history of their article ([what does this mean?](https://track.editorialmanager.com/CL0/https:%2F%2Fjournals.plos.org%2Fplosone%2Fs%2Feditorial-and-peer-review-process%23loc-peer-review-history/1/010f019eaf74f431-9bf2e61a-ce6b-47c6-949d-0c716dfc1c8b-000000/FmQAHH2g57-aSOqeGHBow8GPkG-Sdwy_G2XwvXFaQhY=258)). If published, this will include your full peer review and any attached files.


If you choose “no”, your identity will remain anonymous but your review may still be made public.


**Do you want your identity to be public for this peer review?** For information about this choice, including consent withdrawal, please see our [Privacy Policy](https://track.editorialmanager.com/CL0/https:%2F%2Fwww.plos.org%2Fprivacy-policy/1/010f019eaf74f431-9bf2e61a-ce6b-47c6-949d-0c716dfc1c8b-000000/lrGAnsgucOB0lRw5lZbyd1H1ysJlgSBp240Z1gdRsV0=258).

Reviewer #1: **Yes:** Fernanda Carolina Camargo

Reviewer #2: No
